# Supplementary material for: Measuring Quality of Life in Parents or Caregivers of Children and Adolescents with Celiac Disease: Development and Content Validation of the Questionnaire
Source: Nutrients. 2019 Sep 27;11(10):2302. doi: 10.3390/nu11102302 (PMC6835388; doi:10.3390/nu11102302)
Supplement: Supplementary file 1 [file nutrients-11-02302-s001.pdf]

Table S1. Celiac Disease Patents of Children or Adolescents Quality of Life Questionnaire (CDPCA-QoL)–The Brazilian-Portuguese version Questionário de doença celíaca (QCD)–índice relacionado a qualidade de vida dos pais ou responsáveis de crianças e adolescentes portadores de doença celíaca

1. Com que frequência, no último mês, cuidar da doença **celíaca** do seu filho(a) fez você se sentir cansado(a) ou sem energia?
( ) Sempre
( ) Quase sempre
( ) Algumas vezes
( ) Quase nunca
( ) Nunca

2. Com que frequência, no último mês, você se sentiu irritado(a) e impaciente por causa da doença **celíaca** do seu filho(a)?
( ) Sempre
( ) Quase sempre
( ) Algumas vezes
( ) Quase nunca
( ) Nunca

3. Com que frequência, no último mês, você se sentiu desanimado(a) ou triste por causa da doença **celíaca** do seu filho(a)?
( ) Sempre
( ) Quase sempre
( ) Algumas vezes
( ) Quase nunca
( ) Nunca

4. A doença **celíaca** é uma doença de herança familiar. Com que frequência, no último mês, você se sentiu culpado(a) pela doença **celíaca** do seu filho(a)?
( ) Sempre
( ) Quase sempre
( ) Algumas vezes
( ) Quase nunca
( ) Nunca

5. Com que frequência, no último mês, você se sentiu chateado(a) ou com raiva quando seu filho(a) não seguiu a dieta sem glúten?
( ) Sempre
( ) Quase sempre
( ) Algumas vezes
( ) Quase nunca
( ) Nunca, pois, isso, não me chateia
( ) Nunca, pois, ele segue rigorosamente a alimentação

6. No último mês, você conseguiu relaxar ou se ver livre de qualquer tensão, apesar do seu filho(a) ter a doença **celíaca**?
( ) Nunca
( ) Quase nunca
( ) Algumas vezes
( ) Quase sempre
( ) Sempre

7. No último mês, o quanto você se sentiu capaz em lidar com a dieta do seu filho (a)?
( ) Nunca
( ) Quase nunca
( ) Algumas vezes

( ) Quase sempre
( ) Sempre

8. Nas últimas vezes, que você levou seu filho (a) para consultar, você se sentiu segura e apoiada pelos profissionais de saúde que cuidam da doença **celíaca** do seu filho(a)?

( ) Nunca
( ) Quase nunca
( ) Algumas vezes
( ) Quase sempre
( ) Sempre

9. Com que frequência, você se sente isolado (a) por causa da doença celíaca do seu filho (a), por exemplo, não ter vontade de participar de atividades na casa de outros parentes ou se sentir excluído (a) do grupo?

( ) Sempre
( ) Quase sempre
( ) Algumas vezes
( ) Quase nunca
( ) Nunca

10. Com que frequência, a doença **celíaca** do seu filho (a), tira-lhe a alegria e o otimismo em relação à vida?

( ) Sempre
( ) Quase sempre
( ) Algumas vezes
( ) Quase nunca
( ) Nunca

11. Com que frequência, no último mês, a preocupação com a doença **celíaca** do seu filho(a), tirou-lhe o sono?

( ) Sempre
( ) Quase sempre
( ) Algumas vezes
( ) Quase nunca
( ) Nunca

12. Com que frequência, nos últimos dois meses, você teve dúvidas em permitir que seu filho(a) comesse fora de casa (restaurantes, casa de amigos ou outros familiares) por causa da doença **celíaca** dele?

( ) Sempre
( ) Quase sempre
( ) Algumas vezes
( ) Quase nunca
( ) Nunca

13. Com que frequência, no último mês, você se preocupou com a falta de conhecimento da escola à respeito da doença **celíaca** do seu filho(a)?

( ) Sempre
( ) Quase sempre
( ) Algumas vezes
( ) Quase nunca
( ) Nunca

14. Com que frequência, nos últimos meses, você se preocupou em deixar seu filho (a) aos cuidados de outra pessoa por causa da doença **celíaca** dele(a)?

( ) Sempre
( ) Quase sempre
( ) Algumas vezes
( ) Quase nunca

( ) Nunca

15. Com que frequência, no último mês, você se preocupou com a qualidade dos alimentos sem glúten e com a dificuldade em obtê-los?

( ) Sempre
( ) Quase sempre
( ) Algumas vezes
( ) Quase nunca
( ) Nunca

16. Com que frequência, nos últimos dois meses, você sentiu que o custo com os alimentos sem glúten afetou o seu orçamento ?

( ) Sempre
( ) Quase sempre
( ) Algumas vezes
( ) Quase nunca
( ) Nunca

17. Com que frequência, no último mês, você se preocupou ou teve medo do seu filho (a) vir ater câncer ou outra doença por não seguir bem a dieta sem glúten?

( ) Sempre
( ) Quase sempre
( ) Algumas vezes
( ) Quase nunca
( ) Nunca

18. Com que frequência, no último mês, você teve dificuldade em ler rótulos dos alimentos e identificar se contém glúten?

( ) Sempre
( ) Quase sempre
( ) Algumas vezes
( ) Quase nunca
( ) Nunca

19. Com que frequência, no último mês, você se preocupou com a contaminação cruzada dos alimentos ingeridos pelo seu filho(a) ?

( ) Sempre
( ) Quase sempre
( ) Algumas vezes
( ) Quase nunca
( ) Nunca

20. Com que frequência você sente medo dos resultados dos exames a que seu filho se submete, como exames de sangue ou endoscopia ?

( ) Sempre
( ) Quase sempre
( ) Algumas vezes
( ) Quase nunca
( ) Nunca

21. Com que frequência, no último mês, você sentiu que o cuidar da saúde do seu filho (a) prejudicou o relacionamento com seu parceiro (a)?

( ) Sempre
( ) Quase sempre
( ) Algumas vezes
( ) Quase nunca
( ) Nunca

22. Com que frequência, no último mês, você sentiu que pessoas importantes na vida do seu filho(a) não (seu cônjuge, parentes, etc) não colaboraram com a restrição alimentar dele(a) ?

( ) Sempre
( ) Quase sempre
( ) Algumas vezes
( ) Quase nunca
( ) Nunca

23. Com que frequência, você e sua família recusam convites para participarem de eventos sociais (festas, aniversários) devido a doença **celíaca** do seu filho(a)?

( ) Sempre
( ) Quase sempre
( ) Algumas vezes
( ) Quase nunca
( ) Nunca

24. Com que frequência, a doença **celíaca** do seu filho(a), afeta o planejamento de férias da família ?

( ) Sempre
( ) Quase sempre
( ) Algumas vezes
( ) Quase nunca
( ) Nunca
( ) Não se aplica

25. Com que frequência, você se sente limitado(a) em sua formação profissional ou em sua carreira por causa da doença **celíaca** do seu filho (a) (por exemplo: fazer cursos, assumir outras funções na empresa, etc) ?

( ) Sempre
( ) Quase sempre
( ) Algumas vezes
( ) Quase nunca
( ) Nunca
( ) Não se aplica

26. Com que frequência, no último mês, você sentiu que seus colegas de trabalho ou superiores, demonstraram falta de compreensão em relação à doença **celíaca** do seu filho(a) ?

( ) Sempre
( ) Quase sempre
( ) Algumas vezes
( ) Quase nunca
( ) Nunca
( ) Não se aplica

27. Com que frequência, no último mês, você sentiu que a doença **celíaca** do seu filho (a), afetou sua relação com ele(a) ?

( ) Sempre
( ) Quase sempre
( ) Algumas vezes
( ) Quase nunca
( ) Nunca

28. Nas último mês, o quanto o cuidar da saúde do seu filho(a), fez você sentir que não tem tempo para seus próprios interesses (fazer atividade física, ir ao cabelereiro, sair com amigos, etc) ?

( ) Sempre
( ) Quase sempre
( ) Algumas vezes
( ) Quase nunca
( ) Nunca

29. Nas último mês, o quanto o cuidar da doença **celíaca** do seu filho (a) fez você sentir que não está dando a devida atenção a outros membros da sua família (filhos, cônjuges, etc)?

( ) Sempre
( ) Quase sempre
( ) Algumas vezes
( ) Quase nunca
( ) Nunca

30. Com que frequência, nas últimos dois meses, você sentiu que a preocupação com a doença do seu filho (a) prejudicou a qualidade e a frequência de suas relações sexuais?
( ) Sempre
( ) Quase sempre
( ) Algumas vezes
( ) Quase nunca
( ) Nunca
( ) Não se aplica

Table S2. Celiac Disease Patents of Children or Adolescents Quality of Life Questionnaire (CDPCA-QoL)–The English version

1. How often in the last month did your child’s celiac disease make you feel tired or lacking in energy?
( ) Always
( ) Almost always
( ) Sometimes
( ) Almost never
( ) Never

2. How often in the last month did you feel irritated and impatient due to your child’s celiac disease?
( ) Always
( ) Almost always
( ) Sometimes
( ) Almost never
( ) Never

3. How often in the last month did you feel discouraged or saddened about your child’s celiac disease?
( ) Always
( ) Almost always
( ) Sometimes
( ) Almost never
( ) Never

4. Celiac disease is a genetic disease. How often in the last month did you feel guilty about your child’s celiac disease?
( ) Always
( ) Almost always
( ) Sometimes
( ) Almost never
( ) Never

5. How often in the last month did you feel upset or angry when your child did not follow the gluten-free diet?
( ) Always
( ) Almost always
( ) Sometimes
( ) Almost never
( ) Never

6. In the last month, have you been able to relax or feel stress free, despite your child’s celiac disease?
( ) Never
( ) Almost never

( ) Sometimes
( ) Almost always
( ) Always

7. Over the past month, how often did you feel capable of coping with your child's diet?
( ) Never
( ) Almost never
( ) Sometimes
( ) Almost always
( ) Always

8. The last few times you took your child to medical consultations, did you feel safe and supported by the health professionals who care for your child?
( ) Never
( ) Almost never
( ) Sometimes
( ) Almost always
( ) Always

9. How often do you feel isolated because of your child's celiac disease, for example, not wanting to participate in activities with friends and relatives or feel excluded from groups?
( ) Always
( ) Almost always
( ) Sometimes
( ) Almost never
( ) Never

10. How often does your child's celiac disease take away your joy and optimism?
( ) Always
( ) Almost always
( ) Sometimes
( ) Almost never
( ) Never

11. How often, in the last month, did you have insomnia due to your child's celiac disease?
( ) Always
( ) Almost always
( ) Sometimes
( ) Almost never
( ) Never

12. How often, in the last two months, have you had any doubts about allowing your child to eat out (restaurants, friends' houses, or other family members) because of his or her celiac disease?
( ) Always
( ) Almost always
( ) Sometimes
( ) Almost never
( ) Never

13. How often in the last month have you been concerned about the school's lack of knowledge about your child's celiac disease?
( ) Always
( ) Almost always
( ) Sometimes
( ) Almost never
( ) Never

14. How often in recent months have you been worried about leaving your child in the care of others because of his/her celiac disease?
( ) Always

( ) Almost always
( ) Sometimes
( ) Almost never
( ) Never

15. How often in the last month have you been worried about the quality of gluten-free foods and the difficulties in obtaining them?
( ) Always
( ) Almost always
( ) Sometimes
( ) Almost never
( ) Never

16. How often in the last two months have you felt that the cost of gluten-free food has affected your food budget?
( ) Always
( ) Almost always
( ) Sometimes
( ) Almost never
( ) Never

17. How often in the past month have you worried or been afraid that your child will develop cancer or another disease for not following a gluten-free diet?
( ) Always
( ) Almost always
( ) Sometimes
( ) Almost never
( ) Never

18. How often, in the last month, have you had trouble reading food labels and identifying if they contain gluten or not?
( ) Always
( ) Almost always
( ) Sometimes
( ) Almost never
( ) Never

19. How often in the last month have you been concerned about cross-contamination in your child's food?
( ) Always
( ) Almost always
( ) Sometimes
( ) Almost never
( ) Never

21. How often are you afraid of the tests results when you child undergoes exams such as blood tests or endoscopy?
( ) Always
( ) Almost always
( ) Sometimes
( ) Almost never
( ) Never

22. How often in the last month did you feel that taking care of your child's health harmed your relationship with your partner?
( ) Always
( ) Almost always
( ) Sometimes
( ) Almost never

( ) Never

23. How often in the last month did you feel that important people in your child's life (your spouse, relatives, etc.) did not cooperate with your child's food restriction?

( ) Always
( ) Almost always
( ) Sometimes
( ) Almost never
( ) Never

24. How often do you and your family refuse invitations to attend social events (parties, birthdays) due to your child's celiac disease?

( ) Always
( ) Almost always
( ) Sometimes
( ) Almost never
( ) Never

25. How often does your child's celiac disease affect family vacation planning?

( ) Always
( ) Almost always
( ) Sometimes
( ) Almost never
( ) Never

26. How often do you feel limited in your professional training or career because of your child's celiac disease (for example, taking courses, taking on other duties in the company, etc.)?

( ) Always
( ) Almost always
( ) Sometimes
( ) Almost never
( ) Never

27. How often in the past month have you felt that your co-workers or superiors have shown a lack of understanding of your child's celiac disease?

( ) Always
( ) Almost always
( ) Sometimes
( ) Almost never
( ) Never

28. How often in the last month did you feel that your child's celiac disease affected your relationship with him/her?

( ) Always
( ) Almost always
( ) Sometimes
( ) Almost never
( ) Never

29. In the last month, how much did your child's health care make you feel that you do not have time for your own interests (doing physical activity, going to the hair salon, hanging out with friends, etc.)?

( ) Always
( ) Almost always
( ) Sometimes
( ) Almost never
( ) Never

30. In the last month, how much did caring for your child's celiac disease make you feel that you are not paying attention to other members of your family (children, spouse, etc.)?

( ) Always
( ) Almost always
( ) Sometimes
( ) Almost never
( ) Never

31. How often in the last two months have you felt that concerns over your child's illness has impaired the quality and frequency of your sexual life?

( ) Always
( ) Almost always
( ) Sometimes
( ) Almost never
( ) Never

( ) Does not apply
